# Ruxolitinib Rescues Multiorgan Clinical Autoimmunity in Patients with APS-1

**DOI:** 10.1007/s10875-023-01629-x

**Published:** 2023-12-19

**Authors:** Romain Lévy, Agathe Escudier, Paul Bastard, Coralie Briand, Laura Polivka, Athanasia Stoupa, Cécile Talbotec, Anya Rothenbuhler, Marina Charbit, Dominique Debray, Christine Bodemer, Jean-Laurent Casanova, Agnès Linglart, Bénédicte Neven

**Affiliations:** 1 Laboratory of Human Genetics of Infectious Diseases, Necker Branch, INSERM, Necker Hospital for Sick Children, Paris, EU France; 2 Paris-Cité University, Imagine Institute, Paris, EU France; 3 Pediatric Hematology, Immunology and Rheumatology Unit, Necker Hospital for Sick Children, AP-HP Paris, EU France; 4 St. Giles Laboratory of Human Genetics of Infectious Diseases, Rockefeller Branch, The Rockefeller University, New York, NY USA; 5 Department of Pediatrics, Jean Verdier Hospital, AP-HP, Bondy, EU France; 6 Department of Dermatology, Reference Center for Genodermatoses (MAGEC), Imagine Institute, Necker Hospital for Sick Children, AP-HP Paris, EU France; 7 Pediatric Endocrinology, Gynecology and Diabetology Department, Necker Hospital for Sick Children, AP-HP Paris, EU France; 8 Department of Pediatric Gastroenterology, Necker Hospital for Sick Children, AP-HP Paris, EU France; 9 Department of Endocrinology and Diabetes for Children; Reference Center for Rare Diseases of Calcium and Phosphate Metabolism, Filière OSCAR, ERN BOND, Endo-ERN, Bicêtre Paris Saclay Hospital, AP-HP, Le Kremlin-Bicêtre, EU France; 10 Department of Pediatric Nephrology, Necker Hospital for Sick Children, AP-HP Paris, EU France; 11 Department of Pediatric Hepatology, Necker Hospital for Sick Children, AP-HP Paris, EU France; 12 Howard Hughes Medical Institute, New York, NY USA; 13 Paris Saclay University, INSERM U1185, Bicêtre Paris Saclay Hospital, Le Kremlin-Bicêtre, EU France

**Keywords:** APS-1, AIRE, Ruxolitinib, Jak inhibitor

## Abstract

Autoimmune polyendocrine syndrome type-1 (APS-1) is caused by mono- or biallelic loss-of-function variants of the autoimmune regulator gene *AIRE* underlying early-onset multiorgan autoimmunity and the production of neutralizing autoantibodies against cytokines, accounting for mucosal candidiasis and viral diseases. Medical intervention is essential to prevent or attenuate autoimmune manifestations. Ruxolitinib is a JAK inhibitor approved for use in several autoimmune conditions. It is also used off-label to treat autoimmune manifestations of a growing range of inborn errors of immunity. We treated three APS-1 patients with ruxolitinib and followed them for at least 30 months. Tolerance was excellent, with no medical or biological adverse events. All three patients had remarkably positive responses to ruxolitinib for alopecia, nail dystrophy, keratitis, mucosal candidiasis, steroid-dependent autoimmune hepatitis, exocrine pancreatic insufficiency, renal potassium wasting, hypoparathyroidism, and diabetes insipidus. JAK inhibitors were therefore considered an effective treatment in three patients with APS-1. Our observations suggest that JAK/STAT pathways are involved in the pathogenesis of APS-1 autoimmune manifestations. They also suggest that JAK inhibitors should be tested in a broader range of APS-1 patients.

**Supplementary Information:**

The online version contains supplementary material available at 10.1007/s10875-023-01629-x.

## Introduction

Autoimmune polyendocrine syndrome type-1 (APS-1), also known as polyendocrinopathy-candidiasis-ectodermal dystrophy or APECED, is a monogenic inborn error of immunity (IEI) caused by mono- or biallelic loss-of-function variants of the autoimmune regulator gene *AIRE* [1–4]. APS-1 is a rare condition, with a higher prevalence in Finns, Iranian Jews, and Sardinians (1:9,000 to 1:25,000) due to founder mutations [5, 6]. AIRE is expressed in medullary thymic epithelial cells (mTECs) and mediates the ectopic expression of peripheral tissue-restricted antigens to thymocytes, leading to the negative selection of autoreactive T cells, thereby ensuring “central” immunological tolerance [7]. In patients with APS-1, the thymic escape of autoreactive T cells underlies widespread autoimmunity, with multiple endocrinopathies and autoantibodies (Abs) against interleukin-(IL) 17 [8, 9] and type I interferons (IFNs) [10, 11]. APS-1 is clinically defined as the triad of chronic mucocutaneous candidiasis (CMC), hypoparathyroidism, and adrenal insufficiency. Nevertheless, more than 30 different autoimmune phenotypes have been reported [4–6, 12]. APS-1 typically manifests in early childhood, but new autoimmune manifestations can occur at any age.

The management of patients with APS-1 is challenging [13]. There is no cure and no robust strategy for preventing or attenuating autoimmunity. Current management relies on hormonal substitution treatments for endocrinopathies. Auto-Abs neutralizing IL-17A/F underlie CMC and auto-Abs neutralizing type I IFNs underlie life-threatening COVID-19 pneumonia and other viral diseases [14–16]. Antifungal drugs and antiviral agents are therefore useful, as is ad hoc vaccinations. Diverse immunosuppressive drugs have been used in patient- and disease-based approaches (for autoimmune hepatitis, pneumonitis, or nephritis), with variable efficacy [17, 18]. The range of approved indications for JAK inhibitors (JAKi), such as ruxolitinib and baricitinib, in the management of autoimmune disorders is increasing. Experience with the off-label use of JAKi in the setting of rare diseases, such as IEIs, has been reported, with positive results. These inhibitors have been used in patients with autosomal dominant (AD) STAT1 and STAT3 gain-of-function (GOF) variants, with the JAK-dependent activation of these two STATs providing the rationale for their use in this context [19, 20]. Through analogy with other IEIs, we conducted a pilot study in which we treated three APS-1 patients with ruxolitinib, despite the lack of a clear biological rationale for this approach.

## Methods

Ruxolitinib treatment was prescribed off-label to three adolescents with confirmed APS-1 followed at the Pediatric Immunology-Hematology Department of Necker Children’s Hospital (Paris, France) for alopecia areata (*n* = 2) or steroid-dependent autoimmunity with severe adverse effects (*n* = 1). Ruxolitinib was initiated at a dose of 15 to 20 mg/m^2^/day after the patient (and the parents, for individuals under the age of 18 years) had given informed consent. We then performed a longitudinal observational study with the collection of clinical and biological data.

## Results

### Case 1

Patient 1 was a 17-year-old boy suffering from recurrent urticaria. He presented alopecia areata with an onset at two years of age, onycholysis and intertrigo beginning at six years of age and had had exocrine pancreatic insufficiency since the age of 9 years (Table 1). Compound heterozygous nonsense mutations in *AIRE* confirmed APS-1 at the age of 11 years (c.415C > T / c.1273C > T), when he presented autoimmune hypoparathyroidism, adrenal insufficiency, keratitis and histologically confirmed autoimmune hepatitis (AIH). The treatment initially prescribed comprised azathioprine and steroids for AIH and topical cyclosporine for keratitis, hydrocortisone and 9-alpha fludrocortisone substitution for adrenal insufficiency, calcium plus calcitriol for hypoparathyroidism, pancreatic enzyme replacement and antifungal prophylaxis with fluconazole. This patient also reported chronic fatigue beginning at the age of 13 years, with intense steroid-sensitive and steroid-dependent abdominal pain related to malabsorption and steatorrhea (normal upper and lower gastrointestinal tract histology) with a major impact on his quality of life and the prescription of multiple lines of analgesic medication (amitriptyline, duloxetine, baclofen, oxcarbazepine). Ruxolitinib treatment was initiated at the age of 15 years, at a low dose of 10 mg two times a day (corresponding to 12 mg/m^2^/day), due to comedication with fluconazole. The doses of steroids and azathioprine were tapered and these drugs were discontinued after six and 20 months of ruxolitinib treatment, respectively, with no relapse of AIH. Topical cyclosporine was stopped after 13 months due to the complete remission of keratitis. Intertrigo and onycholysis improved significantly on treatment. Pancreatic enzyme supplementation was tapered, and eventually stopped after 24 months of ruxolitinib, following the disappearance of steatorrhea (from 103 to 6 g/day; Normal range: 1.5–6 g/day), and the normalization of stool elastase levels (from 186 to 719 μg/day; Normal values > 200 µg/day). Calcium supplementation was discontinued after three months. Pain medication was rapidly stopped (Table 1). The spectacular positive effect on many components of the disease led to a remarkable improvement in the patient’s quality of life, as best exemplified by his ability to attend school. The number of treatments prescribed decreased from 12 (> 30 pills a day) before ruxolitinib treatment to five drugs (10 pills) per day after ruxolitinib treatment.

**Table 1:** Characteristics of the treated APS-1 patients, before and after ruxolitinib initiation

Patient	Age at diagnosis (y)	*AIRE* mutation	Age at Ruxo initiation (y)	Ruxo dose, duration (m)	Disease manifestations	Age at onset (y)	Status at Ruxo initiation	Outcome and timing
P1	11	c.415C > T (p.R139X) c.1273C > T (p.Q425X)	15	10 mg BID*, 12 mg/m^2^ (30)	Alopecia areata	2	CR	CR
					Onycholysis and intertrigo	6	active	PR (3)
					Exocrine pancreatic insufficiency	9	active	CR (24)
					Autoimmune hepatitis	11	active**	CR (20) ***
					Hypoparathyroidism	11	active	PR (3)
					Adrenal insufficiency	11	active	stable
					Keratitis	11	active	CR (12)
					Recurrent abdominal pain	13	active	CR (12)
P2	5	c.415C > T (p.R139X) c.967_979del (p.Leu323Serfs*51)	15.5	20 mg BID, 25 mg/m^2^ (25)	CMC	5	active	CR
					Hypoparathyroidism	5	active	PR
					Alopecia areata	10	active	PR (12), CR (24)
					Adrenal insufficiency	7	active	stable
					Renal potassium wasting	13	active	PR
					Diabetes insipidus	12.5	active	CR
P3	7	c.415C > T (p.R139X) c.967_979del (p.Leu323Serfs*51)	19	20 mg BID, 25 mg/m^2^ (31)	Hypoparathyroidism	7	active	PR
					Adrenal insufficiency	8	active	active
					Alopecia areata	12	active	PR (3), CR (12)

*P* patient, *y* years, *CMC* chronic mucocutaneous candidasis, *CR* complete remission, *PR* partial remission, *m *months, *Ruxo *Ruxolitinib; *concomitent treatment with fluconazole; ** on steroids and azathioprine *** discontinuation of steroids (3 m) and azathioprine (20 m)

### Case 2

**Fig. 1:**
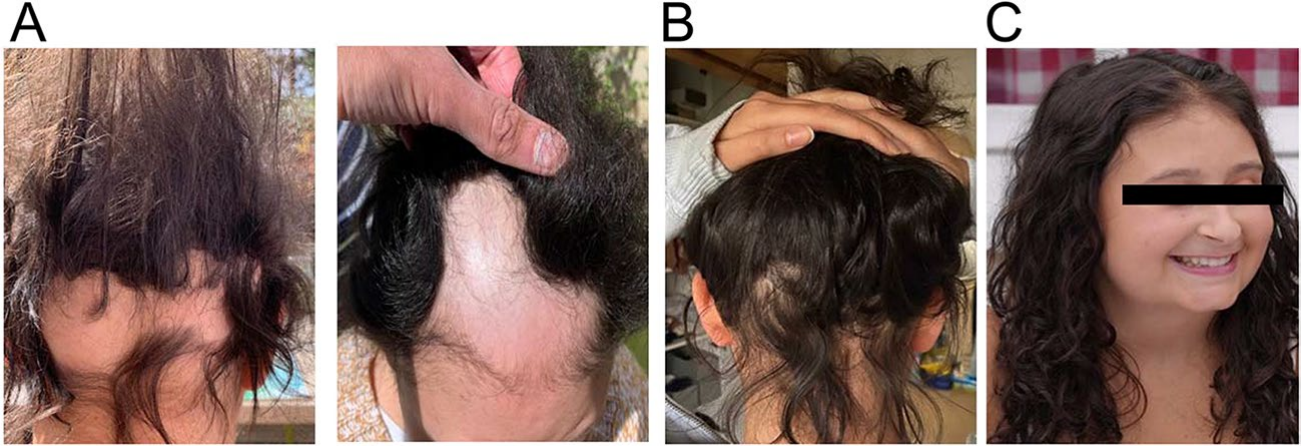
Course of alopecia in Patient 2 Photographs before the introduction of ruxolinitib (**A**) and after 12 (**B**) and 24 (**C**) months of treatment in patient 2

**Fig. 2:**
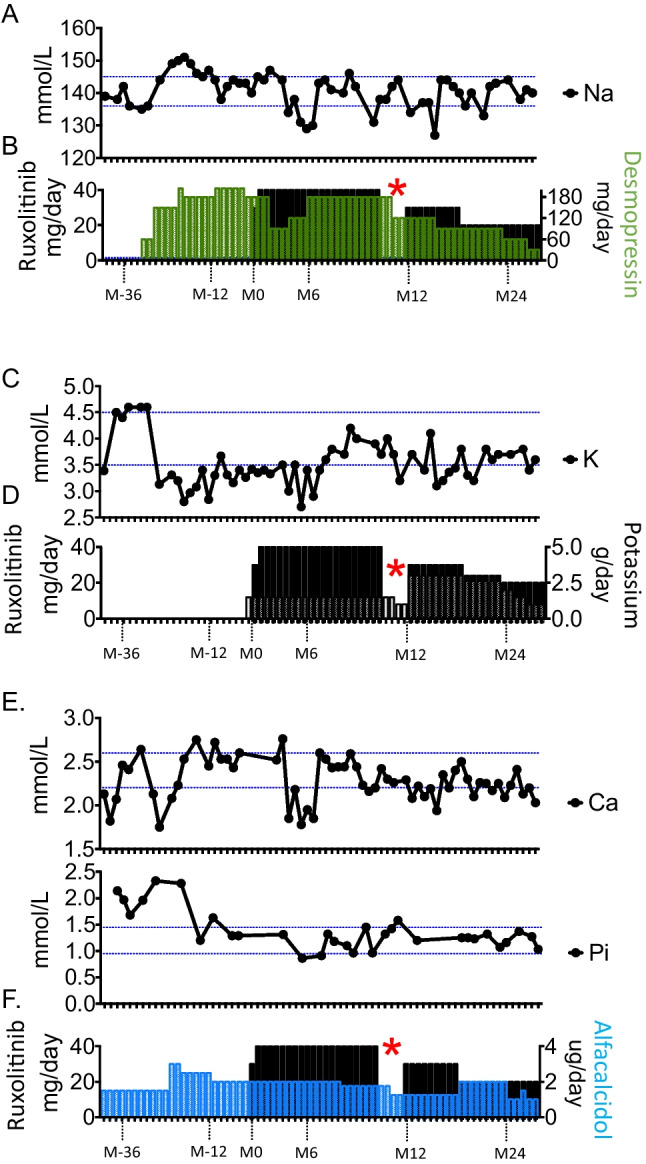
Course of the disease in Patient 2 (**A**-**B**) Course of natremia. Diabetes insipidus was treated by substitution with desmopressin; the treatment and dose are indicated by the green bars (right *y* axis). Note the sodium concentration above or at the upper limit of the normal range (mean ± SD: 144.1 ± 1.18 mmol/L; *n* = 16) before ruxolinitib treatment (black bars) and the recurrent episodes of hyponatremia during the course of ruxolinitib treatment (**A**), suggesting an excess of desmopressin and leading to a gradual decrease of desmopressin dose and the discontinuation of this treatment at the most recent follow-up visit (**B**). Na (blood sodium determination); the reference range is 136–146 mmol/L; the red asterisk indicates the period during which the patient received baricitinib. Follow-up spans of 3.5 years and 2.5 years before and after the initiation of ruxolinitib (R), respectively. (**C**-**D**) Renal potassium wasting was suspected when serum potassium concentration fell below the normal range despite adrenal insufficiency, a condition associated with hyperkalemia. Potassium supplements were prescribed at a dose of up to 3 g/day (empty bars) leading to a slow, but steady increase in blood potassium concentration and a decrease in potassium supplement intake. K (blood potassium determination); the reference range is 3.5–4.5 mmol/L. Follow-up spans of 3.5 years and 2.5 years before and after of the initiation of ruxolinitib, respectively. (**E**–**F**) Hypoparathyroidism was treated with alfacalcidol; treatment and dose are indicated by the blue bars (right *y* axis). Blood phosphate levels decreased on ruxolinitib treatment, from 1.78 ± 0.42 to 1.20 ± 0.18 mmol/L, this decrease being associated with an increase in the percentage of measurements within the normal range from 30 to 91%. Concomitantly, the requirement for alfacalcidol to sustain blood calcium level (calcium 2.35 ± 0.29 and 2.25 ± 0.21 mmol/L; 56 and 62% of measurements in the normal range before and during ruxolinitib treatment, respectively) decreased from 1.9 ± 0.5 to 1.7 ± 0.4 µg/day. Ca (blood calcium determination); the reference range is 2.2–2.6 mmol/L; Pi (blood phosphate determination); the reference range is 0.95–1.45 mmol/L. Follow-up spans of 3.5 years and 2.5 years before and after the initiation of ruxolinitib, respectively

Patient 2 was an 18-year-old female patient who had suffered from oral mucosal candidiasis since the age of five years, when she presented a hypocalcemic seizure revealing hypoparathyroidism (Table 1). *AIRE* gene sequencing showed that she was compound heterozygous for two loss-of-function variants (c.415C > T / c.967_979del). This patient subsequently developed autoimmune adrenal insufficiency, alopecia (Fig. 1A), diabetes insipidus and renal potassium wasting at the ages of 7, 10, 12.5 and 13 years, respectively. Diabetes insipidus was suspected because of a sudden increase in water intake to up to 4 L per day and was confirmed by a water deprivation test and the absence of a neurohypophysis on brain MRI (Supplemental Fig. 1). Desmopressin treatment was initiated, and the management of water, sodium and potassium intakes was challenging due to the combination of adrenal insufficiency, renal potassium leakage and diabetes insipidus. At the age of 15 years treatment with ruxolitinib 10 mg two times a day (12.5 mg/m^2^/day) was initiated for alopecia, with the dose increased to 20 mg two times a day (25mg/m^2^/day) after three months. Hair regrowth was evident after 12 months of treatment (Fig. 1B). After two years of follow-up, the alopecia had fully resolved (Fig. 1C), making it possible to taper the dose to 10 mg two times a day, with the response sustained over an additional six months of follow-up. In addition to improving hair growth, this treatment also improved other manifestations (Table 1). First, despite adequate desmopressin substitution at a dose of up to 160 mg/day, the patient initially complained of persistent hyperdiuresis confirmed by repeated measurements of natremia above or at the upper limit of the normal range (mean ± SD: 144.1 ± 1.18 mmol/L; *n* = 16). Following the introduction of ruxolinitib, she had several episodes of water intoxication, antidiuresis and her natremia decreased significantly (mean ± SD: 139.3 ± 0.83 mmol/L; *n* = 38; *p* = 0.0011) leading to a progressive decrease of desmopressin dose and the cessation of this treatment at last follow-up (Fig. 2A, B). Second, potassium supplements were decreased considerably, from 3 g/day to 0.5 g/day, and this change in dose was accompanied by a lessening of cramps and a progressive increase in serum potassium concentrations (Fig. 2C, D). Third, biochemical hallmarks of hypoparathyroidism improved upon ruxolinitib treatment, suggesting an increase in PTH secretion. Hypoparathyroidism is characterized by hypocalcemia, hyperphosphatemia and an inappropriately low level of parathormone. It is managed by stimulating calcium uptake in the digestive tract with alfacalcidol, a vitamin D analog. Upon ruxolitinib treatment, we observed a significant decrease in serum phosphate levels (from 1.78 ± 0.42 to 1.20 ± 0.18 mmol/L) and a stabilization of serum calcium levels (2.35 ± 0.29 to 2.25 ± 0.21 mmol/L), and alfacalcidol dose was tapered (1.9 ± 0.5 to 1.7 ± 0.4 µg/day) (Fig. 2E, F). Finally, mucosal candidiasis has not recurred since the introduction of ruxolitinib.

### Case 3

**Fig. 3:**
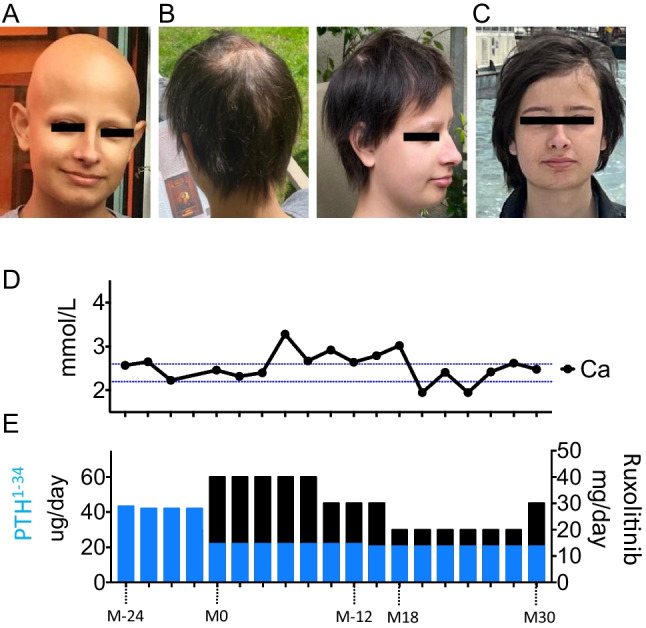
Course of the disease in patient 3 Photographs before the initiation of ruxolinitib (**A**) and after 12 (**B**) and 24 (**C**) months of treatment. (**D**-**E**) Hypoparathyroidism was treated by subcutaneous PTH^1−34^ infusion; the treatment and dose are shown by the blue bars (right *y* axis). While on subcutaneous PTH^1−34^ infusion, the patient experienced several episodes of hypo- and hypercalcemia. Over a period of about two years before the initiation of ruxolinitib, blood calcium levels were adequately maintained within the reference range. Upon ruxolinitib therapy, the PTH^1−34^ requirements for sustaining balanced blood calcium levels decreased from 42.4 ± 0.7 to 21.2 ± 0.7 µg/day. Ca (blood calcium determination); the reference range is 2.2–2.6 mmol/L

Patient 3, a 21-year-old man, the older brother of patient 2, was diagnosed with APS-1 at the age of seven years, due to long-standing symptoms of hypocalcemia caused by hypoparathyroidism (Table 1). Despite treatment with alfacalcidol, the patient suffered repeated episodes of hypo- and hypercalcemia requiring emergency management in hospital. This prompted a switch to a continuous subcutaneous infusion of PTH at the age of 11 years. Acute autoimmune adrenal insufficiency was diagnosed at the age of eight years and has been treated with hydrocortisone and 9-alpha fludrocortisone ever since. At the age of 12 years, this patient developed *alopecia areata universalis* (Fig. 3A), which posed a considerable psychological burden. After careful consideration, ruxolitinib treatment was initiated at a dose of 20 mg two times a day (25 mg/m^2^/day) at the age of 19 years. Hair growth first appeared on the scalp after about three months of treatment, subsequently becoming much stronger. After 12 months of treatment, the alopecia had fully resolved and the patient presented significant eyelash, eyebrow, and beard hair regrowth (Fig. 3B). Ruxolitinib efficacy continued (Fig. 3C), over an additional 19 months of follow-up, and it was possible to taper the dose up to 10 mg two times a day. Remarkably, as reported for his sister, patient 2, this patient presented a decrease in the occurrence of hypo- and hypercalcemia, and a significant decrease in PTH requirements from 42.4 ± 0.7 µg/day to 21.2 ± 0.7 µg/day (*p* < 0.0001) suggesting a partial remission of hypoparathyroidism (Fig. 3D, E).

### Safety

The duration of ruxolitinib treatment was 25, 30 and 31 months for patients 1, 2 and 3, respectively. Overall, the tolerance profile was good, with no infectious adverse events in any of the patients. They all had been infected with varicella zoster virus (VZV) and herpes simplex virus (HSV) during childhood and tested positive for IgG in serological tests performed at the initiation of ruxolitinib treatment. No reactivation of these infections was observed upon treatment. The patients had received all the usual vaccinations, including those for encapsulated bacteria and an annual influenza vaccine booster. All three patients were fully immunized against SARS-CoV-2 before ruxolitinib initiation. Epstein-Barr virus (EBV), cytomegalovirus (CMV) and BK virus were not detected in serum at any point during treatment. P1 and P3 presented mild COVID-19 whilst on ruxolitinib. Patient 2 was briefly switched onto baricitinib after nine months on ruxolitinib, as she presented transient anemia. She was switched back onto ruxolitinib when this condition resolved. Routine biological parameters, including whole blood cell counts, liver and kidney parameters, remained within the normal range.

## Discussion

JAK inhibitors had a beneficial impact on most of the clinical manifestations of the three APS-1 patients treated. Ruxolitinib had a clinically relevant impact on alopecia areata, nail dystrophy, candidiasis, autoimmune hepatitis, hypoparathyroidism, renal potassium leakage, diabetes insipidus, keratitis, and exocrine pancreatic insufficiency. Adrenal insufficiency was the only manifestation that did not respond to treatment, perhaps attesting to the destruction of the cells targeted by the autoimmune process. It is unknown whether this treatment affected the levels of auto-Abs against IL-17 and type I IFNs. Treatment had a positive effect on the patients’ quality of life, with a sustained effect on manifestations after 30 months of follow-up in all three patients. No infectious adverse effects, such as herpes zoster infection, or biological side effects were observed except for transient anemia in patient 2. The dose of ruxolitinib should be adjusted if this inhibitor is prescribed together with drugs inhibiting cytochrome P450 (CYP3A4 and CYP2C9), such as azole antifungal drugs, due to the risk of higher levels of exposure to ruxolitinib leading to adverse effects. This is the first report of the use and efficacy of ruxolitinib in APS-1, and it provides the first documented evidence of an immunosuppressive drug having a broad and positive effect on endocrine components of APS-1. However, this series is too small for strong conclusions to be drawn. Nevertheless, based on these encouraging results, ruxolitinib or other JAK/STAT inhibitors should be tested in more patients with APS-1, including younger patients, before the target tissues are destroyed. A prospective clinical trial is also warranted.

The potential benefits of JAKi in APS-1 patients must be balanced against their potential short- and long-term adverse effects, especially in children. Experience with the off-label use of JAKi to treat other IEIs, such as STAT1 and STAT3 GOF [20, 21], suggests that the safety profile is favorable, but caution is required given the limited number of patients treated and the lack of long-term follow-up. We recently reported our experience of JAKi use in 11 patients with Aicardi-Goutières syndrome; we documented five serious bacterial infections, including two invasive *Streptococcus pneumoniae* infections [22].These invasive pyogenic infections are particularly relevant to APS-1 patients, 10 to 15% of whom have functional asplenia [23, 24]. Furthermore, viral infections, including shingles in particular, are the most frequent complication of JAKi use [25]. Viral infections are particularly relevant in APS-1 patients, who produce auto-Abs neutralizing type I IFNs, increasing the risk of developing severe COVID-19 [15, 16] and herpes viral disease [26]. When considering JAKi prescription for any APS-1 patient, it is essential to check that SARS-CoV-2, influenza, meningococcus and pneumococcus vaccinations are up to date. By contrast, live vaccines such as those against yellow fever, measles, mumps and rubella (MMR), are contraindicated. Other theoretical concerns of a non-infectious nature concerning the use of JAKi in children relate to potential effects on growth and bone metabolism, although there is currently no clinical evidence to support such concerns [27].

Our observations suggest that JAK/STAT signaling plays a key role in the pathogenesis of autoimmune conditions in patients with APS-1. The cellular and molecular pathogenic mechanisms underlying endocrine and non-endocrine tissue damage remain largely unknown. Ruxolitinib, like baricitinib, is a first-generation JAKi, preferentially inhibiting JAK1 and JAK2, but also capable of targeting JAK3 and TYK2, all of these molecules governing leukocytic and non-leukocytic cellular responses to over 50 stimuli [25, 28]. It is not, therefore, reasonable to speculate about possible mechanisms of action within tissues as diverse as those affected in APS-1 patients. Fundamental in-depth studies are required to determine which JAK/STAT pathway should be specifically targeted in APS-1 patients as new generations of more specific JAKi emerge [25]. Such studies will help to determine the most appropriate class of JAKi to use, and which area under the concentration versus time curve (AUC) to target.

### Supplementary Information

Below is the link to the electronic supplementary material.

Supplementary file1 (DOCX 222 KB) Pituitary and brain MRI, Pituitary and brain MRI at the diagnosis of diabetes insipidus in patient 2. T1 sequence. The anterior pituitary is visible within the sella turcica; the hyperdense signal of the neurohypophysis is absent.

## Data Availability

The datasets generated and analyzed during this study are available from the corresponding author on reasonable request.
