# Supplementary figures and images for: Ruxolitinib Rescues Multiorgan Clinical Autoimmunity in Patients with APS-1
Source: J Clin Immunol. 2023 Dec 19;44(1):5. doi: 10.1007/s10875-023-01629-x (PMC10730634; doi:10.1007/s10875-023-01629-x)

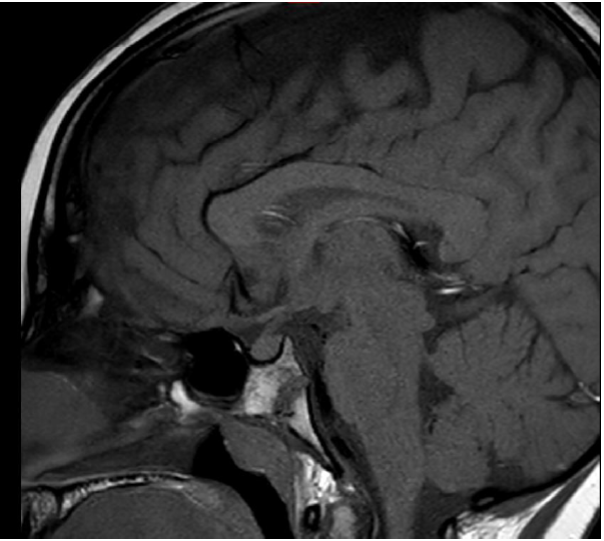

Supplement: Supplementary file 1 — Supplementary file1 (DOCX 222 KB) Pituitary and brain MRI, Pituitary and brain MRI at the diagnosis of diabetes insipidus in patient 2. T1 sequence. The anterior pituitary is visible within the sella turcica; the hyperdense signal of the neurohypophysis is absent. [file 10875_2023_1629_MOESM1_ESM.docx]
